# Robotic pyelolithotomy in a solitary pelvic kidney complicated with fulminant *Clostridium difficile*: a case report

**DOI:** 10.1186/s13256-022-03345-3

**Published:** 2022-03-25

**Authors:** Preston S. Kerr, Nga T. Nguyen, Andrew Martinez, Aditya Srinivasan, Christopher D. Kosarek, Joseph Nicholas Sreshta

**Affiliations:** 1 Division of Urology, The University of Texas Medical Branch at Galveston, 301 University Blvd, Galveston, TX 77555 USA; 2 School of Medicine, The University of Texas Medical Branch at Galveston, Galveston, TX USA

**Keywords:** Robotic surgery, Pyelolithotomy, *C. difficile*, Colitis

## Abstract

**Background:**

Robotic-assisted surgeries have gradually become the standard of care for many procedures, especially in the field of urology. Despite the widespread use of robotic assistance in surgeries, data on its postoperative complications are extremely limited. We detail a rare presentation of fulminant *Clostridium difficile* colitis requiring surgical intervention in a patient with a solitary ectopic pelvic kidney who underwent a robotic-assisted pyelolithotomy. Highlights of the most recent management recommendations for *C. difficile* infection are also presented.

**Case presentation:**

A 26-year-old Caucasian woman who underwent a robot-assisted pyelolithotomy of a pelvic kidney developed tachycardia, leukocytosis, and severe diarrhea 2 days following surgery. Because of her long history of antibiotic use, her severe symptoms were concerning for *C. difficile* colitis. This was confirmed by a *C. difficile* toxin test and a computed tomography scan. She was given recommended antibiotics, but her condition progressively deteriorated. The patient developed fulminant colitis and toxic megacolon, for which she underwent an exploratory laparotomy with subtotal abdominal colectomy and ileostomy creation on the twelfth day of her hospitalization. She fully recovered and was discharged 3 weeks after her subtotal colectomy.

**Conclusion:**

Although robotic surgeries have been shown to have several advantages, risk of postsurgical complications remains. We present a rare case of fulminant *C. difficile* colitis that complicated a robotic-assisted pyelolithotomy. Active prevention, early detection, and optimization of management are essential to preventing unfavorable outcomes.

## Background

The use of robotic-assisted surgery (RAS) has grown extensively since its introduction 30 years ago, especially in urological procedures. The implementation of RAS provides many benefits, including smaller incisions, decreased blood loss, shorter hospital stays, and lower incidence of surgical complications. Nonetheless, all the complications of open or laparoscopic surgery are still present in RAS [1, 2]. We present a case of robotic-assisted pyelolithotomy in a patient with an ectopic pelvic kidney complicated by postoperative development of fulminant *C. difficile* colitis progressing to toxic megacolon.

*C. difficile* is one of the most common nosocomial pathogens. In recent years, there has been an increase in both the incidence and severity of *C. difficile* infections (CDI) [3]. However, a review of the current literature revealed no prior reports of fulminant CDI after robotic pyelolithotomy in a pelvic kidney or robotic surgeries in general. Owing to the uniqueness of this patient’s presentation and clinical course, we believe this case will better enable clinical and surgical practitioners to prevent, identify, and treat *C. difficile*-related complications in similar patients.

## Case presentation

**Fig. 1:**
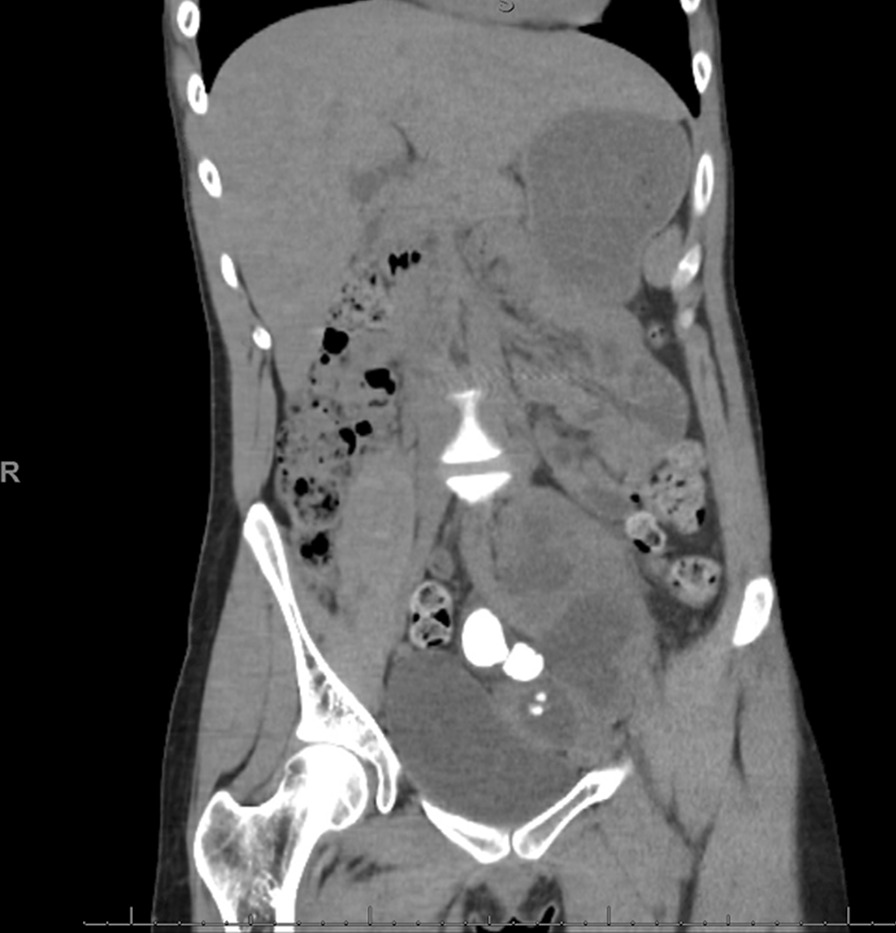
Coronal slice of a computed tomography abdomen/pelvis showing a large stone burden in a solitary pelvic kidney

**Fig. 2:**
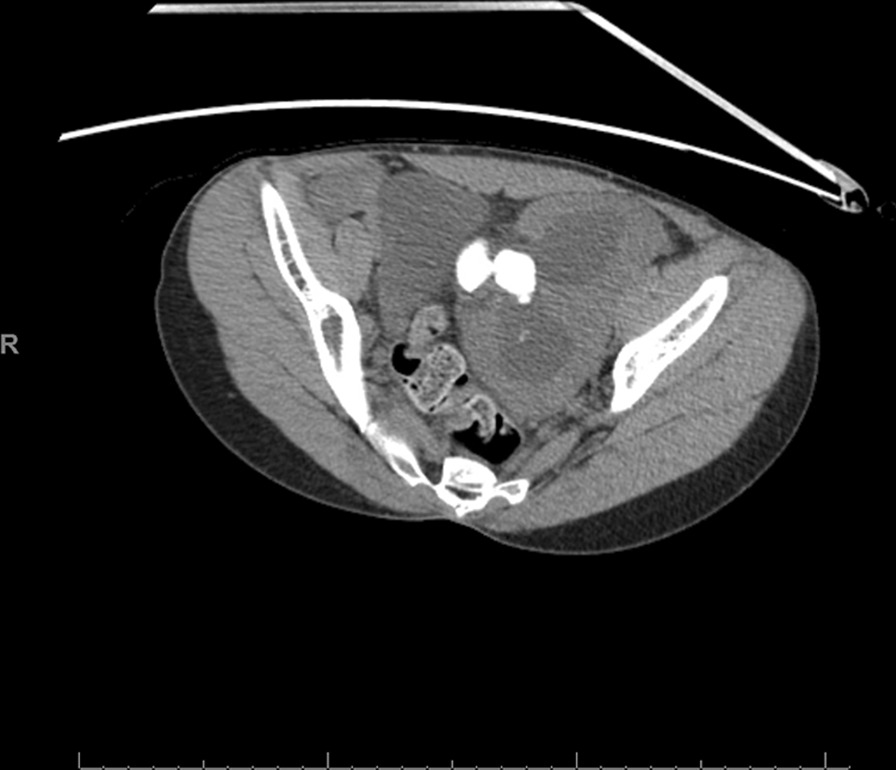
Axial slice of a computed tomography abdomen/pelvis showing a large stone burden in a solitary pelvic kidney

A 27-year-old Caucasian woman with a past medical history of solitary pelvic kidney and recurrent urinary tract infections (UTI) presented for her scheduled robotic-assisted pyelolithotomy. Nine weeks prior to the procedure, she had presented to the emergency department (ED) with unrelenting left flank pain and nausea without fever or lower urinary tract symptoms. She noted that she had had repeat infections over the last few years requiring antibiotics, but never had a complete workup owing to a lack of insurance. A computed tomography (CT) scan of the abdomen and pelvis showed an enlarged solitary left pelvic kidney with severe hydronephrosis, perinephric stranding, and multiple large stones in the renal pelvis measuring up to 2.7 cm (Figs. 1 and 2). She underwent urgent percutaneous nephrostomy (PCN) tube placement with no complications. Urine culture at that time was negative. She was discharged on a 10-day prophylactic regimen of trimethoprim–sulfamethoxazole. After obtaining indigent care funding, she was seen in clinic where a percutaneous nephrolithotomy was discussed; however, given her unique anatomy, she was scheduled for a robotic-assisted pyelolithotomy.

Two weeks prior to her scheduled robotic-assisted pyelolithotomy, she returned to the ED for fever, chills, left lower quadrant abdominal pain, and bleeding from her nephrostomy bag. She was noted to be febrile at 38.7 °C and tachycardic up to 119 beats per minute. Her voided urinalysis was positive for nitrites and various inflammatory markers, concerning for a urinary tract infection. A CT of the abdomen and pelvis at that time demonstrated scattered multifocal patchy hypoattenuation throughout the renal cortex suggestive of cortical scarring and/or infection. She was admitted with a diagnosis of acute pyelonephritis and was treated with empiric intravenous ceftriaxone. Final urine cultures noted pan-sensitive *Serratia marcescens* and *Pseudomonas aeruginosa*. She was discharged on a 7-day regimen of cefpodoxime. No subsequent eradication cultures were obtained.

Eight days after her discharge, she underwent a robotic-assisted pyelolithotomy without complications. At the conclusion of the case, the patient had only a double-J ureteral stent and her PCN was removed. On her second postoperative day, the patient was tolerating a regular diet, ambulating, and passing flatus. She reported mild suprapubic abdominal pain. We noted she was tachycardic in the low 100s but otherwise afebrile and hemodynamically stable. This was initially attributed to pain. She was subsequently started on piperacillin and tazobactam after her intraoperative urine culture returned positive for *P. aeruginosa* later that day.

**Fig. 3:**
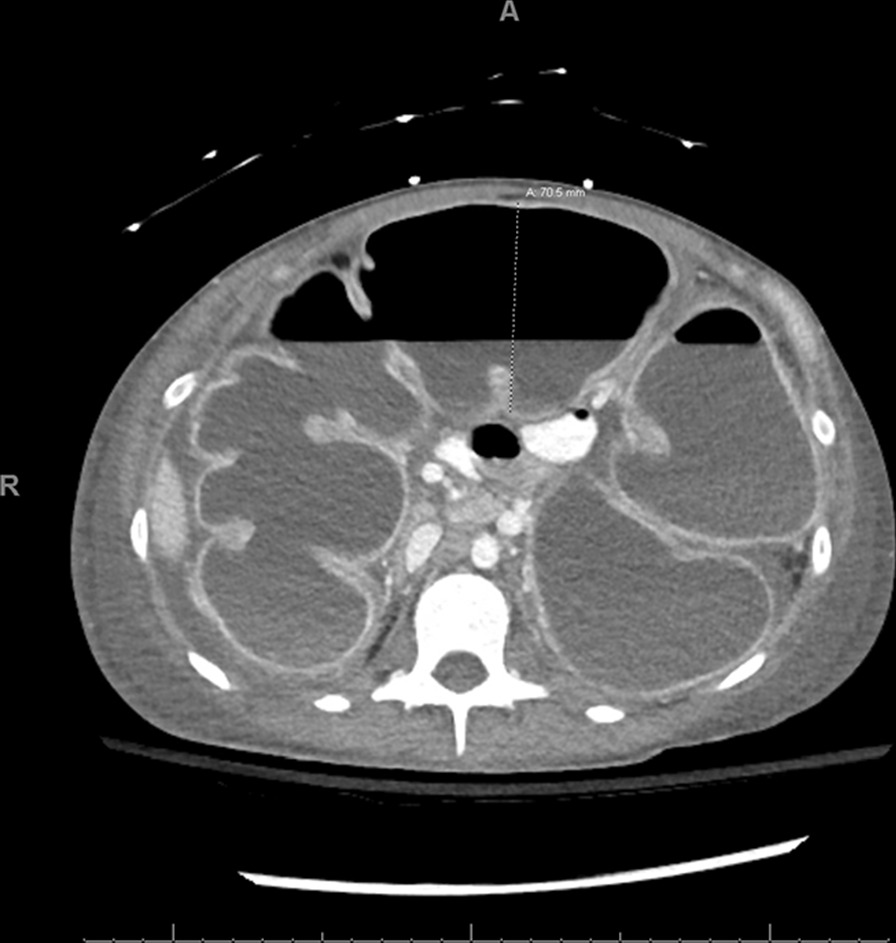
Axial slice of a computed tomography abdomen/pelvis showing dilated large bowel with partial enhancement of the wall of the colon, suggestive of colitis

**Fig. 4:**
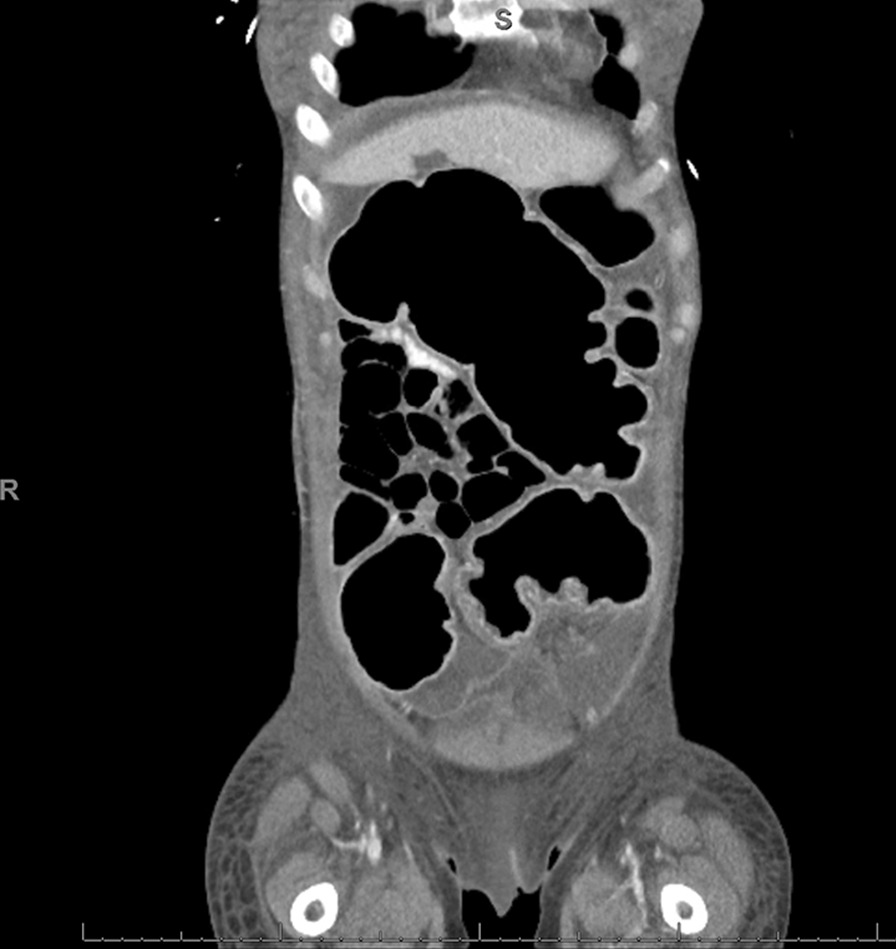
Coronal slice of a computed tomography abdomen/pelvis showing dilated large bowel with partial enhancement of the wall of the colon, suggestive of colitis

On her third postoperative day, the patient remained hemodynamically stable and continued to meet postoperative milestones, including passing flatus, but she had persistent tachycardia in the 130s. An electrocardiogram noted only sinus tachycardia. Her abdominal examination remained unchanged and benign. The sensitivity of the intraoperative urine culture returned highly susceptible to fluoroquinolones, and we transitioned her to oral ciprofloxacin. On the fourth postoperative day, she continued to have tachycardia now as high as 180s and began exhibiting abdominal distension. She also reported multiple frequent watery bowel movements. She continued to exhibit intermittent hypotension despite administration of intravenous fluids. Orthostatic vitals were negative. Lab work now revealed mild leukocytosis at 14,000 with a left shift. A CT scan of the abdomen and pelvis showed dilation of the large bowel and partial enhancement of the wall of the colon, suggestive of colitis (Figs. 3 and 4). Cardiology was consulted owing to the profound tachycardia, which they attributed to sepsis.

The patient was transferred to surgical intensive care unit (ICU). Though she had no prior history of *C. difficile* infections, her recent fluoroquinolone use, CT findings, and overall clinical presentation were strongly suggestive of sepsis secondary to *C. difficile* colitis; thus, a *C. difficile* stool toxin test was conducted. Aside from a possible *C. difficile* infection, early concerns arose that the patient may have had an incompletely treated UTI or an infected intraabdominal fluid collection. As a result, ciprofloxacin was stopped and ertapenem was started. Additionally, a regimen of oral and rectal vancomycin was initiated. Despite these measures, the patient continued to have tachycardia with rising leukocytosis and intermittent fever. On postoperative day 6, her *C. difficile* toxin returned positive and she was started on additional intravenous metronidazole. While in the ICU, she was managed by an interdisciplinary team led by surgical intensivists, including infectious disease, internal medicine, cardiology, general surgery, and nutrition.

On postoperative day 8, repeat abdominopelvic imaging showed worsening of her diffuse colonic dilation with diffuse mucosal enhancement and mural thickening. Fidaxomicin was added to the antibiotic regimen, and the oral vancomycin dosage was increased. Her abdominal examination continued to show distension and mild tenderness to palpation in all quadrants. On postoperative day 12, she remained largely unchanged and a repeated CT abdomen and pelvis showed a distension of the entire colon measuring up to 8 cm at the transverse colon with bowel wall edema. This was consistent with toxic megacolon (Fig. 1). On the following day, the patient underwent an exploratory laparotomy with a subtotal colectomy and ileostomy creation. Two days after the surgery, the patient was extubated and remained hospitalized for a postoperative management complicated by malnutrition, bilateral pleural effusions, and persistent tachycardia. She was discharged 3 weeks after her colectomy to a rehabilitation facility. Her ureteral stent placed at the time of surgery was removed at her hospital follow-up with urology. She is currently awaiting a reversal of her ileostomy by colorectal surgery.

## Discussion

The introduction of RAS has drastically changed the platform of minimally invasive surgery. Compared with the traditional laparoscopic approach, RAS not only offers shorter durations of surgical procedures but also allows greater dexterity, wider range of control, easier intracorporeal suturing, and three-dimensional visualization with a faster learning curve. Additionally, RAS has been shown to be associated with fewer complications, such as bleeding, morbidity, and readmission [4]. As a result, RAS has produced promising results in a wide range of procedures, especially those in the field of urological surgery. While utilization of RAS has become the gold standard in the treatment of prostate cancer, it is now being utilized more frequently in selected cases of urolithiasis and ureteric reconstruction. However, RAS has not been used extensively in patients with congenital pelvic kidneys complicated by urolithiasis.

The incidence of ectopic kidneys is between 0.3 and 0.5 per 1000 [5]. Owing to the abnormal placement of the kidney, ureteropelvic junction obstruction and stones are very common. Unlike orthotopic kidneys, the access to pelvic kidneys is made difficult, both anteriorly and posteriorly. These challenges can be overcome with the use of the robotic system, which improves access, simplifies intracorporeal reconstruction, and expedites stone extraction. As with all other procedures, robotic pyelolithotomy carries the standard surgical risks. Currently, the literature is sparse, with only a few case reports describing robotic pyelolithotomy in ectopic pelvic kidneys, and data on its potential complications are very limited [6, 7].

Infection is one of the most common postoperative complications. Among several infectious causes, *C. difficile* has been shown to be the most common organism causing hospital-acquired infection, and its burden is increasing in surgical patients. *Clostridium difficile* infection is associated with extended hospitalizations, extra costs, and risk of readmission, which become a considerable burden to both patients and hospitals [3, 8]. The highest rate of CDI has been reported after lower-extremity amputation (2.6%), bowel resection (0.9%), and gastric or esophageal operation [3]. However, its occurrence in urological procedures, especially those utilizing robot-assisted systems, has not been well described. To our knowledge, this is the first reported case of CDI on a robotic pyelolithotomy in a pelvic kidney.

With the rapid rate of antibiotic resistance in *C. difficile* and the challenges in its management, postsurgical CDI has increased in incidence and severity and become a major health issue worldwide. A fulminant course can develop in severe CDI and is associated with great morbidity and mortality. Therefore, it is important to remain up to date with current management guidelines. According to guidelines by the World Society of Emergent Surgery (WSES), unnecessary antibiotic agent(s) and proton pump inhibitors should be discontinued if CDI is suspected [9]. Empirical therapy is generally avoided if there is low suspicion for CDI. However, if CDI is strongly suspected, empirical therapy can be initiated while awaiting test results. The determination between pharmacological and surgical intervention largely depends on the severity of the disease, and this is detailed in the most recent 2021 Infectious Diseases Society of America (IDSA) Guidelines [10]. For nonsevere CDI, defined as having diarrhea with a white blood cell count < 15,000 cells/mL and/or serum creatinine <1.5 mg/dL, oral fidaxomicin or vancomycin is recommended. For severe disease states that exceed the above criteria, oral fidaxomicin is preferred, and the treatment regimen may be extended to 14 days. This patient’s clinical course took place in 2020, prior to the most recent publication of IDSA guidelines, and it is generally accepted that metronidazole has fallen out of favor as a first- or second-line agent. Recurrent CDI is optimally managed with fidaxomicin with possible adjunctive use of bezlotoxumab [10]. Surgical management should be considered in patients with fulminant colitis who progress to systemic toxicity, indicated by changes in mental status. For patients with fulminant colitis, emergent colectomy improves survival over continued medical management [11]. Factors that have been shown to strongly correlate with surgical mortality in CDI include age 70 years or older, severe leukocytosis or leukopenia (white blood cell count, ≥ 35,000 µL or < 4000 µL) or bandemia (neutrophil bands, ≥ 10%), cardiorespiratory failure (intubation or vasopressors), dialysis dependence, chronic obstructive pulmonary disease, and wound class III [12]. Mortality is also affected by the specialty that takes care of these patients. It has been shown that patients who are cared for by surgical specialties have higher survival rates than those in nonsurgical departments [13, 14]. In addition to pharmacological and surgical management, supportive care is vital. Intravenous fluid resuscitation, albumin supplementation, and electrolyte replacement should be provided to all patients with severe CDI [9, 14].

As a knee-jerk reaction, her postoperative hypotension and tachycardia were thought to be sepsis secondary to UTI, and hence, CDI was not detected at this early state. As a result, multiple antibiotics including piperacillin–tazobactam, ciprofloxacin, and ertapenem were utilized during her course. In a recent analysis of the US Food and Drug Administration adverse event reporting system, all of the aforementioned antibiotics were strongly associated with CDI [15]. Additionally, as recommended by the WSES and IDSA guidelines, because of her multiple concomitant antibiotics, fidaxomicin should have been added earlier in her hospital course.

In this case, pyelolithotomy was originally delayed owing to the patient’s financial situation, which led to multiple recurrent episodes of urinary tract infections. In an attempt to treat the UTI, several antibiotics were used, which increased the drug resistance and subsequent risks for multiple postsurgical complications. Because of the long history of antibiotic use, she was at an increased risk for developing CDI, which should have been considered as a leading differential diagnosis earlier in the patient’s clinical course. As a result, this case presentation helps emphasize the importance of maintaining continuity of care and effectively utilizing financial resources that are available to our patients. These can make a vast impact in patients’ health outcomes, especially in cases complicated by financial difficulties.

## Conclusion

Robot-assisted systems have been shown to have several advantages over the traditional laparoscopic approach. However, current data on their postsurgical complications are still limited. CDI after robotic surgery remains a rare but potentially life-threatening complication. As a result, active prevention and timely management of CDI in the surgical patient are critical.

## Data Availability

Not applicable.

## Abbreviations

RAS: Robotic-assisted surgery
CDI: *C. difficile* infections
UTI: Urinary tract infection
ED: Emergency department
CT: Computed tomography
ICU: Intensive care unit
WSES: World Society of Emergent Surgery
